# Fog–Haze Transition and Drivers in the Coastal Region of the Yangtze River Delta

**DOI:** 10.3390/ijerph19159608

**Published:** 2022-08-04

**Authors:** Rui Lyu, Wei Gao, Yarong Peng, Yijie Qian, Qianshan He, Tiantao Cheng, Xingna Yu, Gang Zhao

**Affiliations:** 1 Shanghai Key Laboratory of Atmospheric Particle Pollution and Prevention (LAP3), Department of Environmental Science and Engineering, Fudan University, Shanghai 200438, China; 2 Shanghai Meteorological Bureau, Shanghai 200030, China; 3 Department of Atmospheric and Oceanic Sciences & Institute of Atmospheric Sciences, Fudan University, Shanghai 200438, China; 4 Institute of Eco-Chongming (SIEC), Shanghai 200062, China; 5 Shanghai Qi Zhi Institute, Shanghai 200232, China; 6 Innovation Center of Ocean and Atmosphere System, Zhuhai Fudan Innovation Research Institute, Zhuhai 518057, China; 7 Key Laboratory for Aerosol-Cloud-Precipition of China Meteorological Administration, Nanjing University of Information Science and Technology, Nanjing 210044, China; 8 Yunnan Meteorological Service Center, Kunming 650034, China

**Keywords:** fog, haze, aerosol, CCN, meteorological condition

## Abstract

Low-visibility events (LVEs) are severe weather phenomena that are closely linked with anthropogenic pollution, which negatively affects traffic, air quality, human health, and the environment. This study conducted a two-month (from October to December 2019) continuous measurement campaign on Chongming Island in Shanghai to characterize the LVEs transition and its drivers. The LVEs accounted for 38% of the time during the campaign, of which mist accounted for 14%, fog–haze for 13%, haze for 6%, and fog for 5%. The fog and mist mainly occurred from midnight to early morning, while haze mostly occurred during the daytime. Different LVEs were interdependent and transitioned from one to another. Fog generally turned into haze after sunrise, while haze turned into fog after sunset. Their formation and evolution were caused by the combined impacts of meteorological conditions and aerosol particles. It was found that temperature difference was the dominant meteorological factor driving the evolution of LVEs. Within the short term, cooling led to a greater increase in relative humidity than humidification. Radiative cooling during the night promoted the formation of fog and mist. During fog and mist events, cloud condensation nuclei (CCN) were mainly internally mixed due to the impact of fog droplet removal and aqueous/heterogeneous aerosol reactions occurring under high humidity. Increased CCN concentration appeared to increase the fog droplet number and liquid water content in fog events. Overall, conditions of high humidity and high particle loading were conducive to LVEs, whereas conditions of sufficient water vapor at a low particle level and sufficient particles at a low humidity level also caused LVEs. This study provided insights into LVEs classification, evolution scheme, and aerosol roles from a micro point of view. The findings could be useful for improving forecasts of local radiative fog and other LVEs.

## 1. Introduction

Low visibility events (LVEs) associated with air pollution have occurred frequently and widely in China in recent decades, posing adverse effects on traffic and human health [1,2]. LVEs are becoming more frequent in China due to air pollution that has increased since the 1980s, reaching a peak in the early 21st century. The Chinese government has made significant investments to significantly to mitigate the air pollution problem since 2013 [3]. Although air quality has improved and the number of haze days has been significantly reduced through strict implementation of control measures, dense fog events have become more frequent in recent years [4], and the coexistence and transformation of fog and haze frequently occur within a short period of time. This makes the characteristics and causes of LVEs a complex issue in the context of effective emission reduction.

LVEs are attributed to the synergistic impacts of anthropogenic emissions and meteorological conditions in and around urban regions [5,6,7,8]. Up until now, the formation, development, and dissipation of individual or isolated LVEs in time and space have been investigated in various aspects by previous studies [9,10,11,12,13,14,15]. Nevertheless, different types of LVEs such as fog, mist, haze, and fog–haze often are interdependent and can be transformed by each other depending on varying environmental conditions. The understanding of the evolution of LVEs and even interaction remains limited because of sparse long-term comprehensive measurements. Ambient conditions provide favorable atmospheric background and play important roles in the formation and maintenance of LVEs, such as humidity, temperature, wind, planetary boundary layer (PBL), atmospheric circulation, and other factors [16,17,18,19]. High relative humidity and aerosol abundance can stimulate and accelerate particle hygroscopic growth, which has been a proven important contributor to intensifying light extinction and atmospheric visibility reduction [20,21,22]. The forces of atmospheric circulation are very complex and vary with terrain at different geographical locations [23,24]. In eastern China, the advection of warm and wet air masses driven by the southerlies has been found to encourage fog occurrence, but the weakened northerlies inhibited aerosol transportation to accumulate local particle matter in the cold seasons [2,25].

Soluble aerosols such as sulfate, nitrate, and sea salt, and coarse aerosols such as dust, which can act as CCN, have important contributions to cloud/fog formation and precipitation [26,27,28,29], and is a breakthrough in understanding the transformation of LVEs from a microscopic perspective. CCN activation is generally affected by aerosol size distribution [30], chemical composition [31,32], aging [33], and mixing state [34]. Although particle size has been announced as the major factor in determining aerosol CCN activity [35], at low supersaturation (SS) levels, particle chemical composition also plays a significant role in aerosol activation [36], especially in terms of fog droplet formation.

In order to explore the evolution of LVEs, we selected Chongming Island for a field observation site, located in the marginal intersection of the ocean and the Yangtze River Delta (YRD) region, which was readily subjected to abundant anthropogenic pollutants and sufficient water vapor. The previous method to classify low visibility weather using average daily visibility and relative humidity made it difficult to distinguish LVEs at the time scale of less than one day and capture weak LVEs and LVEs transitions such as the coexistence of haze–fog events [37,38,39]. This study took measures to resolve LVEs on an hourly time scale to understand the evolution of LVEs and related their driving factors from a microscopic viewpoint. This paper attempts to investigate the haze–fog transition processes induced by shifting aerosols and meteorological conditions and the role of aerosols using high-temporal resolution data. The results improve the refined prediction accuracy of atmospheric visibility and fog.

## 2. Data and Methodology

### 2.1. Observation Site and Measurements

**Figure 1:**
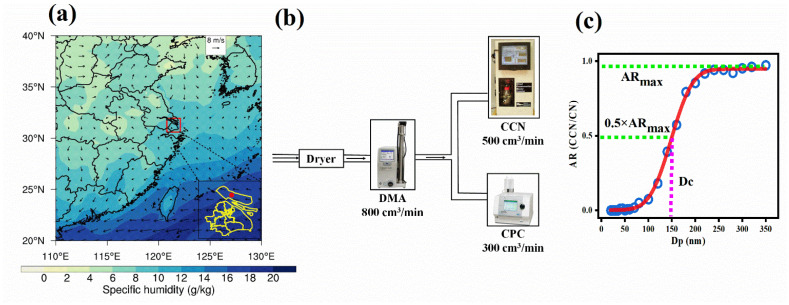
(**a**) The averaged 850 hPa wind field and 10 m specific humidity from 0:00 to 7:00 (LT) on 2 November 2019. The red dot at the bottom right is the field experimental site. (**b**) The instrumental set-up. (**c**) Measured (circle) and fitted size-resolved particle activation ratio curves.

Autumn and winter were affected by particulate matter pollution and stable weather conditions, which were the seasons having a high incidence of LVEs and were often accompanied by the mutual transition of LVEs. The field campaign was conducted from October through December (122 days) in 2019 at the Meteorological Observation Station on Chongming Island of Shanghai (31°39′ N, 121°30′ E, Figure 1). Meteorological data with 1 h resolution were provided by the Shanghai Meteorological Bureau. Hourly wind field and vertical profile of temperature were from the second Modern-Era Retrospective analysis for Research and Applications (MERRA-2) reanalysis dataset at a 0.5° × 0.625° resolution [40].

CCN number concentration was measured by a single-column continuous-flow streamwise thermal gradient CCN counter (CCN-100, DMT Inc., Boulder, CO, USA) at six discrete supersaturation (ss) levels (0.1–1.0%). The 500 cm^3^/min airflow was divided into sample air and sheath air in accordance with 1:10 ratio. More details of CCN counter were described elsewhere [41,42].

One set of Scanning Mobility Particle Sizer (SMPS) was employed to measure size-resolved particle number concentrations within 10–350 nm, consisting of Long Differential Mobility Analyzer (DMA Model 3082, TSI Inc., Shoreview, MN, USA) and Condensation Particle Counter with 300 cm^3^/min flow (CPC Model 3776, TSI Inc., Shoreview, MN, USA). As shown in Figure 1b, the CPC and CCN counters were connected in parallel, and the dried air sample entered the DMA at a rate of 800 cm^3^/min and was then split into two streams to enter the CPC and CCN counters.

The Fog Monitor (FM-120, DMT Inc., Boulder, CO, USA) was used to measure in situ the real-time size distributions of 2–50 μm fog droplets with a sampling rate of 1 m^3^ min^−1^. Fog droplet number concentration (Nd), liquid water content (LWC), and effective diameter (ED) were computed using the measured droplet spectrum.

### 2.2. LVEs Classification and Meteorological Data

With the development of meteorological modernization, manual observations have been replaced with automatic acquisition instruments. Atmospheric visibility (VIS) has been gradually shifted from manual to machine observations since 2013 in China. The meteorological optical range (MOR) observed by the automatic Forward Scattering Visibility Meter, is defined as the path length in atmosphere required to reduce the luminous flux in a collimated beam, which is emitted from an incandescent lamp at a color temperature of 2700 K, to 5 percent of its original value [43,44]. However, the optical-based MOR is not strictly equal to the human eye-based visual range (VR) because of the visual threshold of human eyes [45,46]. The ratio of MOR to VR is about 3:4 based on Lambert-Bouguer Law and Koschmieder Law [47]. The VIS used in this paper were machine measurements and were revised according to the following equation.

(1) $${\mathrm{VIS}}_{corr}=\mathrm{VIS}\times \frac{4}{3}$$

Atmospheric visibility criteria of less than 10 km have been commonly recognized to define LVEs. Based on international organization regulations and aerosol physicochemical characteristics, Wu et al. [48,49] proposed two RH ranges of >95% and <80% for fog and haze, respectively, which were also widely recognized and used by current weather services [39,50]. In this paper, the LVEs, including mist, fog, fog–haze and haze weather, were identified and classified at each observational time based on RH and VIS_*corr*_ (Table 1) by our previous structured method and processing flows [2]. When VIS < 1 km and RH ≥ 95%, it was defined as a fog event. When 1 km < VIS ≤ 10 km and RH ≥ 95%, it was defined as mist (light fog) event. When VIS < 10 km and RH < 80%, it was defined as a haze event. When VIS < 10 km and 80% ≤ RH < 95%, it was defined as fog–haze event. To reduce the uncertainty, the visibility reduction caused by precipitation has been excluded from this study.

As Klein and Hartmann [51] demonstrated, low-tropospheric stability (LTS), defined as the potential temperature difference between 700 hPa and surface, is an indicator of temperature inversion strength, or static stability of the lower troposphere. The variation of LTS was more closely related to the annual cycle of PBL height than the variation of near-surface temperature [52]. LVEs, unlike low clouds, developed mainly in the boundary layer, so the 850 hPa potential temperature was employed here. LTS was calculated as the difference between the potential temperature at 850 hPa and the surface,

(2) $$\mathrm{LTS}=\Delta \theta =\theta_{850}-\theta_{\mathrm{surface}}$$

where higher LTS generally indicates a stable lower troposphere. Moreover, specific humidity (q) describes air moisture content, which were calculated from pressure, temperature, and relative humidity [53].

### 2.3. CCN Efficiency Spectra and Hygroscopicity Parameter

Aerosol activated ratio (AR) is defined as the ratio of CCN number concentration (N_CCN_) to condensation nuclei number concentration (N_CN_). Aerosol CCN activity spectrum is fitted with a cumulative Gaussian (normal) distribution function using a non-linear least-squares fitting routine with three parameters (Figure 1c),

(3) $$f_{N_{\mathrm{CCN}}/N_{\mathrm{CN}}}=a\left(1+\mathrm{erf}\left(\frac{D_{p}-D_{c}}{\sigma \sqrt{2}}\right)\right)$$

where erf is error function, *a* is half the maximum value of *f*_N_CCN_/N_CN__, *D_p_* is particle diameter, *D_c_* is the critical diameter at *f*_N_CCN_/N_CN__ equal to a. *σ* is the standard deviation of cumulative Gaussian distribution function that can be regarded as the indicator for the extent of CCN external mixing [54]. The heterogeneity of particle chemical composition can be represented by the ratio of σ and *D_c_* (*σ*/*D_c_*) [55].

The parameter kappa (κ) has been generally used to characterize the hygroscopicity of aerosol particles [55]. The κ-Köhler equation can be represented as [29]:

(4) $$S_{k}\left(D\right)=\frac{D^{3}-D_{p}^{3}}{D^{3}-\left(1-\kappa \right)D_{p}^{3}}exp\left(\frac{A}{D}\right)$$

where *S_k_* is saturation ratio, *D* is droplet diameter, *D_p_* is the diameter of the dry aerosol particle. κ is the hygroscopicity parameter of aerosol particles, which is calculated from the critical saturation ratio (*S_c_*) and critical diameter (*D_c_*) from the following equation:

(5) $$\kappa =\frac{4A^{3}}{27D_{c}^{3}{\left(\ln S_{c}\right)}^{2}}$$

(6) $$A=\frac{4\sigma_{w}M_{w}}{RT\rho_{w}}$$

where *ρ*_*w*_ is the density of pure water (about 997.04 kg m^−3^), *M*_*w*_ is the molecular weight of water (0.018 kg mol^−1^), *σ*_*w*_ is the surface tension of solution–air interface, which is assumed to be the value of pure water (0.0728 N m^−1^), *R* is the universal gas constant (8.314 J mol^−1^K^−1^), *T* is the temperature in Kelvin (298.15 K), and *D*_c_ is the critical diameter (m).

## 3. Results and Discussion

### 3.1. Overview of Targeted LVEs

A series of LVEs occurred on Chongming Island in October and December of 2019. Mist occurred in 65 days, and fog occurred in 26 days of 122 days. In addition, fog–haze occurred in 62 days, and haze occurred in 40 days. The frequency of LVEs classified on a 1 h time scale was 38.08% during the observation period, which was dominated by mist (14.22%), followed by fog–haze (13.22%), haze (5.75), and fog (4.89)%. Besides LVEs, rain accounted for 7.97% and clean events accounted for 53.94%. Table 2 shows the averages of various parameters for different weather conditions. The mean VIS and RH, respectively, were 15.09 km and 76.74% during the observation period. Fog events had the lowest VIS of 0.41 km and the highest relative humidity of 97.37%. RH was only 63.24% during haze events, and VIS (7.03 km) was mainly attributed to particles rather than water vapor. During fog–haze events, the N_CN_ was up to 15,259 cm^−3^, which was consistent with early observations of 15,000 cm^−3^ during haze episodes in Shanghai [23] (Leng et al., 2016) and 14,967 cm^−3^ during winter in Guangzhou [56] (Duan et al., 2018). The N_CCN_ increased along with the SS and was related well to the N_CN_.

**Figure 2:**
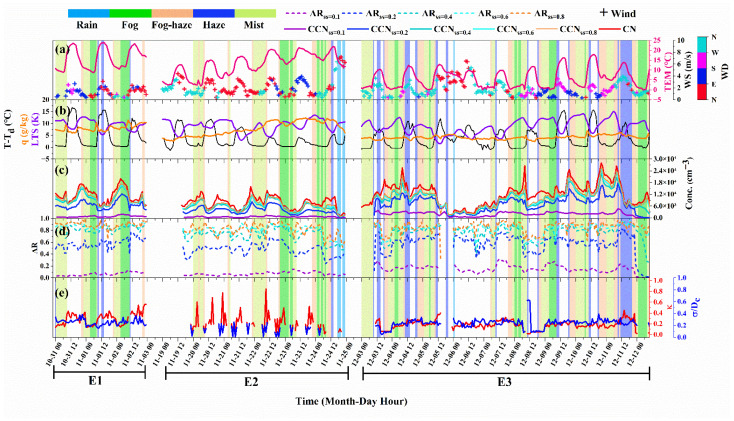
Time series of hourly-averaged (**a**) temperature (TEM), wind direction (WD) and wind speed (WS), (**b**) low tropospheric stability (LTS) and specific humidity (q), (**c**) number concentrations of condensation nuclei (CN) and cloud condensation nuclei (CCN), (**d**) aerosol activated ratio (AR) and (**e**) hygroscopicity (κ) and chemical heterogeneity (*σ*/*D_c_*) of particles during three typical low-visibility event periods (E1: 31 October to 2 November, E2: 19 November to 24 November and E3: 3 December to 12 December). The background colors refer to the type of weather conditions.

Figure 2 depicts the time series of meteorological and microphysical parameters during LVEs periods. Overall, LVEs always showed large particulate matter content and high hygroscopicity, and they transitioned and evolved with time but rarely existed independently. Generally, fog and mist events occurred at night, especially from midnight to early morning, with an average temperature of 7.58 °C and 6.82 °C, respectively. Haze usually occurred in the daytime with an average temperature of 12.42 °C, while fog–haze usually occurred before and after fog or mist events with an average temperature of 8.55 °C. The wind direction in the event of fog or mist was generally northwesterly with wind speeds less than 2 m/s. During haze and fog–haze events, however, more than 35% of winds were greater than 3 m/s, and the north and northwest winds were predominant.

### 3.2. Characteristics of LVEs

#### 3.2.1. Thermodynamic Situation

**Figure 3:**
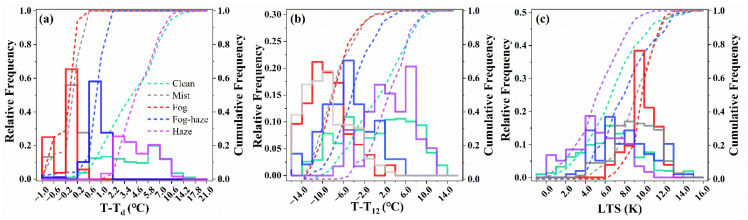
The probability distribution functions of (**a**) air-dewpoint temperature difference (T-T_d_), (**b**) 12 h temperature gradient (T-T_12_), and (**c**) low tropospheric stability (LTS, up to 850 hPa). The solid line is the relative frequency, and the dashed line is the cumulative frequency.

Figure 3 depicts the probability distributions of air-dewpoint temperature difference (T-T_d_), 12 h temperature gradient (T-T_12_), and LTS during LVEs. T-T_d_ requirements for fog and mist formation were the most restrictive. A bimodal distribution of fog and mist occurring between −0.2 and 0.2 °C was observed. The first peak, in which fog and mist occur at 65% and 56%, stretched between −1.0 and −0.6 °C, while the second peak was located between −1.0 and −0.6 °C. Fog–haze T-T_d_ presented a unimodal distribution within 0.6–1.0 °C, with a peak frequency of 58%. The T-T_d_ frequency spectrum of haze events varied between 1.0 and 10.6 °C. T-T_d_ of 0.2 °C could be considered as the threshold for separating fog–haze and fog/mist events, whereas T-T_d_ of 2.2 °C could be used for separating haze and fog–haze events.

The radiation cooling of fog and mist can be quantified using T-T_12_. T-T_12_ showed distinct features according to weather types as well. About 94% of the fog and mist events had negative T-T_12_ values, mostly less than −8 °C, providing evidence of the impact of terrestrial energy release on the formation of radiation fogs at night [57,58]. The T-T_12_ of the fog–haze events were mostly between −6 and −4 °C, with a maximum frequency of 21%, indicating that fog–haze was partly influenced by radiation cooling. When haze occurs, it usually occurred during the daytime, and its corresponding T-T_12_ was almost always positive (0–8 °C). When fog occurred, frequencies tended to concentrate at LTS larger than 9 K (79%), showing higher stability than other LVEs. LTS for haze events was relatively small, with an average of 6.24 K, even less than for clean events (7.63 K). The stable atmosphere restrained the vertical mixing of particulate matter and water vapor, conduced to gather particles and droplets within the boundary layer, and then formed haze or fog events and maintained them. 

Previous studies have also revealed that increased atmospheric stability allowed particles to accumulate near the surface and caused air pollution in the North China Plain (NCP) and the YRD [9,59]. In addition, the interactions of particles with PBL enhanced the atmospheric inversion and contributed to the worsening and prolonging of haze and fog events [60,61]. However, in this study, haze events occurred more often under unstable conditions during the field campaign, revealing a significant contribution of external transport of aerosols from inland. The observation site was greatly influenced by the pollutants from Jiangsu province in the northerly wind and from the urban area of Shanghai in the southerly wind (Figure S1).

#### 3.2.2. Microphysical Properties

**Figure 4:**
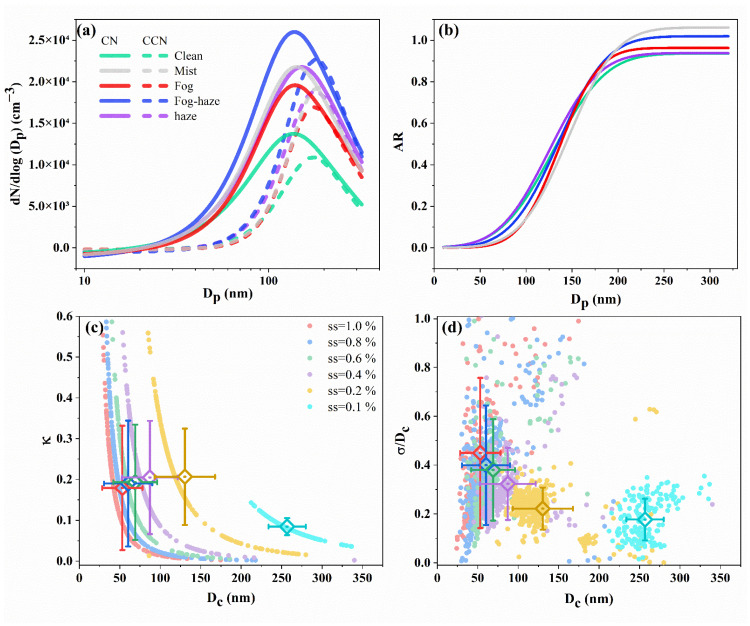
The averaged (**a**) size distributions of condensation nuclei (CN) and cloud condensation nuclei (CCN) and (**b**) size-resolved particle activation ratio. (**c**) Particle critical activation diameter (*D_c_*) vs. aerosol hygroscopic growth parameters (κ) and (**d**) heterogeneous parameters (*σ*/*D_c_*) derived from CCN activation curves at different supersaturation (SS). The error bar represents the standard deviation of parameters at different SS.

Figure 4a,b present the averaged size distribution of condensation nuclei (CN) and CCN number concentrations (N_CN_ and N_CCN_) and CCN activation curve under various atmospheric conditions. The N_CN_ and N_CCN_ of the fog–haze event were the highest among all types of LVEs, followed by haze, mist, and fog, which suggested the removal effect of the fog droplets on particulate matter [62]. The particle activation curves of fog and mist increased steeply with particle diameter (*D_p_*), while the activation curves of haze and clean period showed relatively flat patterns, inferring stronger inhomogeneity of aerosol population and higher external mixing degree in the latter [63]. The activation curve steepness of the fog–haze event was moderate but displayed a high aerosol activation degree, which was second only to mist.

Figure 4c,d shows the relationships of aerosol hygroscopic growth factor (κ) and heterogeneity parameter (*σ*/*D_c_*) with particle critical activation diameter (*D_c_*). As for aerosol hygroscopicity, the dual effects of particle size and water vapor on aerosol activation were described in classic Köhler theory [64], that was, at given supersaturation (SS), the larger the particle critical size, the smaller the hygroscopicity. On the other hand, at a given particle critical size, the higher the supersaturation, the smaller the hygroscopicity. The variability of aerosol activated fraction has been believed to mostly depend on particle size, while the aerosol composition is of secondary importance [35,65]. Ovadnevaite et al. [30] also pointed out that the surface tension of particles prevails over the solute effect due to organic compounds in cloud droplet activation. In terms of aerosol heterogeneity (*σ*/*D_c_*), the internal mixing particles (e.g., aged particles) with ignorable heterogeneity (zero) were preferentially activated at low SS levels., The particle heterogeneity increased at high SS levels, indicating that favorable water vapor conditions allowed external mixing particles (e.g., low soluble particles) with different compositions emitted from various sources to grow and even enhance CCN loadings. As shown in Table 2, the highest average κ (SS = 0.2%) value was measured to be 0.23 in haze events, which could be affected by secondary inorganic species formation [66], and the lowest κ value was 0.15 in fog events probably due to the removal of fog droplets to high hygroscopic aerosols. At the beginning of fog or mist, the easily-activated particle population finished activation and growth preferentially, and then they were in-fog removed by droplets through nucleation scavenging and collision-coalescence, leaving the remainder remained inactivated [62,65]. It was also worth noting that the lower *σ*/*Dc* values usually occurred during fog and mist events and hinted at intensive internal mixing of particles compared to other events. This result was slightly different from the previous study in LinAn [63], likely due to stronger heterogeneous or/and liquid-phase reactions contributing to the production of secondary aerosol species [56,67].

#### 3.2.3. Fog Microstructure

**Figure 5:**
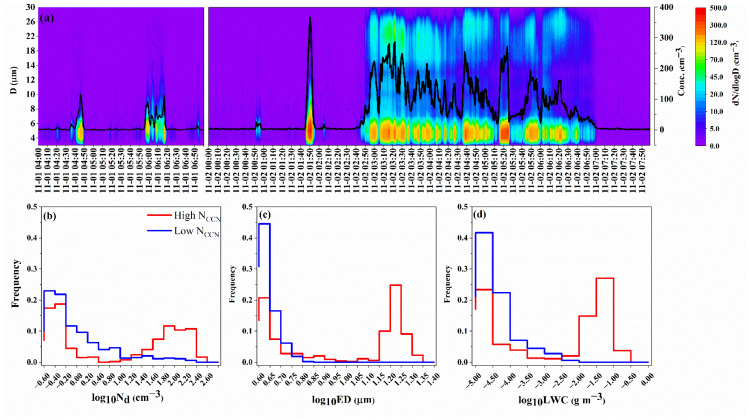
Temporal variations of (**a**) fog droplet concentration and size distribution on 1 and 2 November and (**b**–**d**) probability distribution of the fog droplet concentration (N_d_), equivalent diameter (ED), and liquid water content (LWC) for the high N_CCN_ (>75th percentile) and low N_CCN_ cases (<25th percentile).

Several fog weather processes were captured by FM-120 during the field campaign. The temporal variations of the fog-droplet size distributions on 1 and 2 November are depicted in Figure 5a. In the early stages of fog events, small fog droplets gradually grew into large droplets through collision and coalescence [65]. There existed a dynamic competition for fog droplet evolution among activation, growth, and removal. In particular, the collision-coalescence process reduced fog droplet number exponentially, and the large fog droplets were removed by gravity easily. Moreover, the latent heat released from water vapor condensation enhanced tiny turbulent disturbance and increased the frequency of water vapor exchange, which was conducive to droplet diffusion and fog development but unconducive to fog maintenance due to water vapor dissipation [68].

Known as a general rule of cloud physics, given almost invariant or limited LWC in clouds, the entrained aerosols can increase the concentration of droplets [65,69,70]. Like a cloud, fog can also trap particulate pollutants from environmental air near the surface. The samples with Nccn that were lower than the 25th percentile and greater than the 75th percentile were classified as “low CCN” and “high CCN” cases, respectively, to highlight the influence of CCN on fog microphysical characteristics (Figure 5b–d). Compared with the low CCN case, more fog droplets appeared in the high CCN case. It was noteworthy that the fog droplets’ LWC increased with CCN. A similar phenomenon was demonstrated in marine stratocumulus clouds over the southeastern Pacific Ocean [71,72] and in warm clouds over Eastern China [73,74]. The ED of the fog droplets increased unexpectedly along with their number in the high CCN case. Under high CCN conditions, the ED of the fog droplets increased along with their number, which was due to enough small fog droplets colliding with each other to promote the formation of large droplets, which was verified in the bimodal structure of the fog droplet size spectrum in Figure 5a. This result implied the marked effect of aerosols on fog droplets and fog evolution in addition to meteorological conditions.

### 3.3. LVEs Evolution and Driving Factors

#### 3.3.1. Footmark of LVEs Evolution

**Figure 6:**
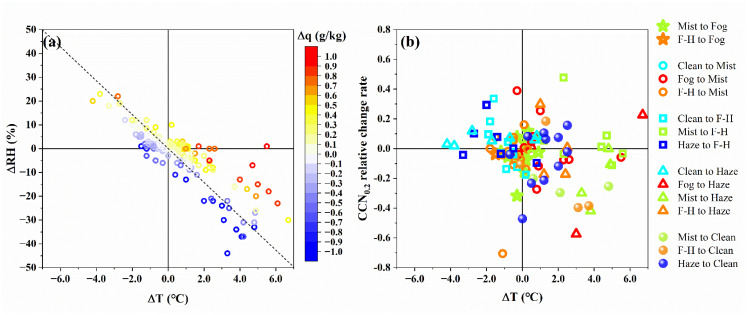
(**a**) Response of relative humidity change (ΔRH) to temperature change (Δ*T*) and specific humidity (Δq) change during the evolution of LVEs. (**b**) Scattering of temperature difference (Δ*T*) vs. CCN_0.2_ relative change rate in the evolution of LVEs. The abbreviation for fog–haze event is F-H.

It can be seen from Figure 2 that the LVEs have mostly interdependent and coexisting relationships throughout the campaign, and this relationship constantly changed from day to night. Temperature, wind speed, atmospheric stability, and aerosol concentration consistently showed significant temporal variations, acting in concert to influence the evolution of LVEs. To analyze in detail the driving factors of the evolution of LVEs, the changes in meteorological and aerosol parameters were calculated for each LVEs transition (Table S1). The N_CN_ increased by 5.96% when a clean event turned into the haze, while N_CCN_ increased by 9.63% when a clean event turned into the fog–haze. In addition, it was found that the CCN reduction (−1454 cm^−3^) was more significant than that of CN (−1271 cm^−3^) when fog turns to haze, supporting the role of the fog droplets in the scavenging of CCN [57,62]. The temperature has been identified as the most important meteorological variable in the evolution of LVEs because it caused the strongest changes in relative humidity when compared to other variables. Figure 6a illustrates the effects of temperature and specific humidity changes on the relative humidity in the transition of LVEs. It was found that Δ*T* caused a greater change in relative humidity than Δq. Temperature decrease was more likely to cause RH to increase than a humidity increase, and temperature increase was also more likely to cause RH to decrease, even with a strong humidity increase. Usually, the rate of change of *T* exceeds that of q by an order of magnitude (Table S1). Even in the case of strong water vapor transport, it was necessary to reduce the difference between the specific humidity and the saturation specific humidity by cooling. As the Δq increased, the time to reach ambient saturation by cooling would be shortened. Consequently, Δ*T* should be given more attention for short-term fog/mist variations and forecasting. Figure 6b shows the relationship between the Δ*T* and CCN change rates during LVEs transition. The transition from clean to LVEs was accompanied by an increase in CCN and/or a cooling process, while the transitions from fog, fog–haze, and haze events to clean periods were accompanied by a significant increase in temperature and/or decrease in CCN.

**Figure 7:**
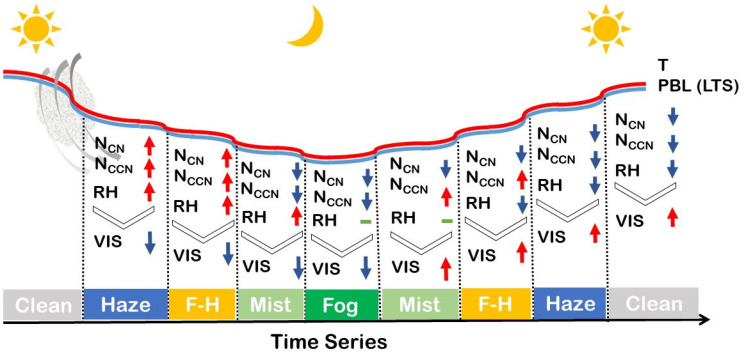
Conceptual model showing how the low-visibility events evolve with time.

Figure 7 shows a conceptual model of the ideal evolution of LVEs. Most haze or fog–haze events occurred before or after sunset and were associated with aerosols transported from external sources. The decreasing temperature and boundary layer height then caused an increase in relative humidity and an accumulation of particulates near the surface. With rising aerosol hygroscopic growth, atmospheric visibility decreased rapidly, and at the same time, droplets and dry particles coexisted to form fog–haze events. Through condensation and absorption of water vapor, more CCN became droplets, and the fog–haze events turned into mist or fog events, resulting in a decrease of 0.96 km or 4.99 km in visibility, respectively. The increasing temperature resulted in the fog event transitioning to mist or haze after sunrise. In the afternoon, fewer LVEs occurred due to the increasing height of the boundary layer and turbulence enhancing the diffusion of pollutants.

#### 3.3.2. Mechanism of LVEs Formation and Evolution

According to Figure 2, we selected three episodes from 31 October to 2 November (E1), 19 to 24 November (E2), and 3 to 12 December (E3) in order to examine the evolution of LVEs. Both particulate matter and environmental humidity were increased during E1, which was influenced by northeasterly winds (Figure S1). This promoted the formation of LVEs extremely easily, especially for fog and mist. LVEs occurred even at relatively low particle loadings during E2 under the condition of large aerosol hygroscopicity and specific humidity. During E3, even in cases of low ambient humidity and low hygroscopicity of aerosols, the large particulate matter loadings carried by the westerly winds also contribute to abundant activation of CCN in the LVEs. LVEs were known to be triggered and maintained by a combination of meteorological and aerosol conditions, but our results suggested they could also be triggered by any one of these two when they were extremely prevalent. 

**Figure 8:**
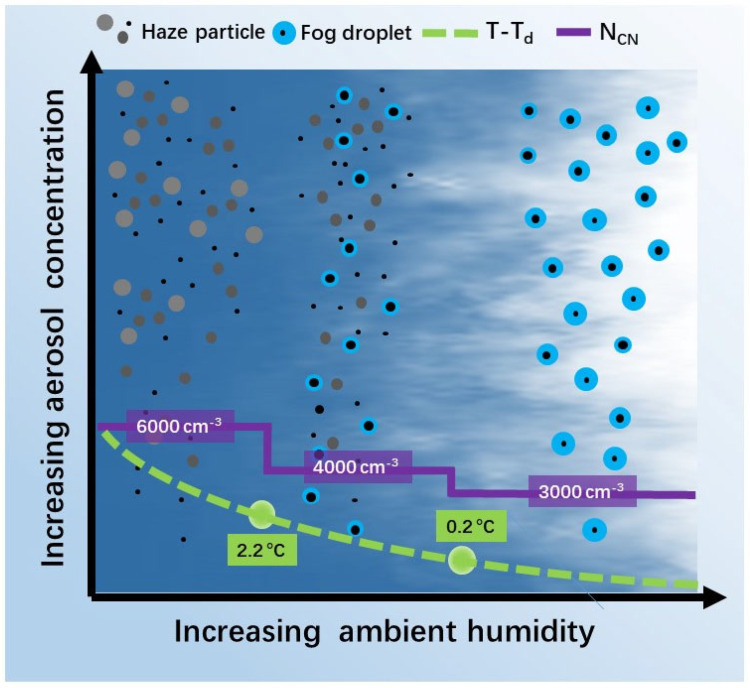
Conceptual model showing how the low-visibility events evolve with different meteorology and aerosol conditions.

Figure 8 illustrates how the LVEs developed with these two drivers. All LVEs benefited from increased aerosol concentration, while fog events were promoted by increased ambient humidity. Mist or fog formed when T-T_d_ dropped below 0.2 °C, and N_CN_ rose to 3000 cm^−3^, while haze formed when T-T_d_ exceeded 2.2 °C and the N_CN_ rose twice (6000 cm^−3^). The value presented here is based on the statistical analysis of this study. A larger number of field campaigns were needed to investigate whether this threshold was universal for regions and seasons beyond Shanghai. However, this conceptual model and threshold will be extremely valuable in understanding the formation, evolution, and transition of LVEs.

## 4. Conclusions

Shanghai Chongming had been plagued by sporadic or continuous LVEs throughout the winter of 2019. A field campaign was conducted to investigate the LVEs from October to December of that year. The previous method to classify low visibility weather using average daily visibility and relative humidity made it difficult to distinguish LVEs at a time scale of less than one day and capture weak LVEs and LVEs transitions, such as the coexistence of haze–fog events. This study took measures to resolve LVEs on an hourly time scale and better understand the evolution of LVEs and their related driving factors from a microscopic viewpoint.

The total frequency of hourly low-visibility events was as high as 38.08%. Different LVEs existed interdependently, among which fog–haze events with relatively high frequency (13.22%). LVEs exhibited significant diurnal variations, which could be explained by the variation in temperature, wind speed, low tropospheric stability, and aerosol concentration. Fog and mist events mostly occurred at night, especially from midnight to early morning, whereas haze events mostly occurred in the daytime. More than 35% of the haze events had wind speeds greater than 3 m/s in Chongming Island, in which the north wind contributed the most, indicating that haze in this region was mostly caused by long-distance transported aerosols from inland. For fog and mist events, the surface radiation cooling effect was significant at night. At the same time, the lower boundary layer height inhibited the vertical diffusion of water vapor at night, which accumulated near the ground and formed radiation fog.

In terms of microphysical properties, as the aerosols hygroscopicity increased, the inhomogeneity of aerosols gradually decreased. However, the aerosols in fog and mist events showed relatively low hygroscopicity and a high degree of internal mixing due to the removal effect of high hygroscopicity particles by fog droplets and the heterogeneous or/and liquid-phase reactions of aerosols under high humidity, respectively. The number concentration, effective diameter, and liquid water content of the fog droplets were more remarkable in high CCN conditions than in low CCN levels.

The formation and evolution of LVEs were the results of the joint impact of anthropogenic pollution and meteorological conditions. High ambient humidity and particle concentrations were certified to operate LVEs; otherwise, higher water vapor at low particle level (or higher particle concentrations at low humidity level) was also a trigger mechanism. The temperature difference was the most critical meteorological driving factor in the evolution of LVEs. Cooling was more likely to improve the ambient saturation than humidification in the short term. Fog or mist was triggered when T-T_d_ dropped to 0.2 °C, and NCN reached 3000 cm^−3^, while when *T* was greater than 2.2 °C, NCN needed to rise twice (6000 cm^−3^) to form haze.

Questions surrounding how atmospheric physical and chemical processes can affect the formation and evolution of LVEs merit further exploration. In the future, we will estimate the contribution of individual driving factors to LVEs, and focus on the influence of aqueous reactions and the aerosol–droplet–meteorology interactions on the evolution of LVEs.

## Figures and Tables

**Table 1:** Key criterion for different low-visibility events and frequency of occurrence in all weather types during October–December 2019.

Event	Relative Humidity	Corrected Visibility	Frequency
Mist	≥95%	>1 km and ≤10 km	14.22%
Fog	≥95%	≤1 km	4.89%
Fog–haze	≥80% and <95%	≤10 km	13.22%
Haze	<80%	≤10 km	5.75%

**Table 2:** Average of various parameters in different weather conditions.

	Total	Clean	Mist	Fog	Fog–Haze	Haze
VIS (km)	15.09 ± 11.20	22.60 ± 8.78	3.88 ± 2.53	0.41 ± 0.28	6.14 ± 2.26	7.03 ± 2.14
RH (%)	76.74 ± 19.16	67.13 ± 17.78	96.60 ± 0.95	97.37 ± 0.79	88.80 ± 4.35	63.24 ± 12.87
Q (g/kg)	6.31 ± 2.23	6.26 ± 2.23	6.09 ± 2.16	6.66 ± 2.77	6.33 ± 2.33	5.75 ± 2.25
WS (m/s)	2.16 ± 1.40	2.53 ± 1.25	0.86 ± 0.55	0.60 ± 0.45	1.77 ± 1.43	2.58 ± 1.18
TEM (°C)	11.25 ± 5.81	13.14 ± 5.43	6.82 ± 4.87	7.58 ± 6.29	8.55 ± 5.01	12.42 ± 5.38
T-Td (°C)	4.18 ± 4.15	6.08 ± 4.18	0.34 ± 0.40	0.17 ± 0.37	1.66 ± 0.84	6.61 ± 2.94
T-T_12_ (°C)	−0.11 ± 7.05	2.54 ± 6.70	−7.18 ± 4.29	−6.98 ± 4.05	−3.13 ± 4.54	5.08 ± 4.41
LTS (K)	8.26 ± 2.98	7.63 ± 3.09	10.08 ± 2.15	10.77 ± 1.34	9.05 ± 2.70	6.24 ± 2.50
N_CN_ (cm^−3^)	10,693.89 ± 5040.45	8149.76 ± 3384.94	12,425.72 ± 4558.25	11,126.61 ± 4283.45	15,259.92 ± 5999.67	12,531.21 ± 4598.33
N_CCN0.1_ (cm^−3^)	1282.21 ± 1035.00	910.52 ± 888.90	1419.81 ± 914.10	1352.83 ± 1043.03	1743.94 ± 987.62	2122.98 ± 1218.47
N_CCN0.2_ (cm^−3^)	6320.10 ± 3524.22	4550.97 ± 2522.37	7323.24 ± 2875.43	6581.53 ± 3776.09	9219.85 ± 3991.85	7817.80 ± 3388.98
N_CCN0.4_ (cm^−3^)	8182.53 ± 4295.94	6024.05 ± 3032.78	9619.13 ± 3656.28	8784.11 ± 3874.93	12,444.96 ± 5010.90	9955.04 ± 3779.64
N_CCN0.6_ (cm^−3^)	8886.53 ± 4491.39	6645.27 ± 3054.92	10,464.37 ± 3843.57	9375.86 ± 3848.87	13,412.57 ± 5413.48	10,450.91 ± 4142.75
N_CCN0.8_ (cm^−3^)	9574.63 ± 4666.29	6832.19 ± 3281.54	11,456.41 ± 3518.29	10,374.32 ± 3696.18	13,751.17 ± 5582.00	10,818.45 ± 4405.55
AR_0.1_	0.05 ± 0.07	0.05 ± 0.07	0.04 ± 0.06	0.06 ± 0.08	0.06 ± 0.07	0.08 ± 0.09
AR_0.2_	0.44 ± 0.26	0.42 ± 0.27	0.42 ± 0.24	0.44 ± 0.28	0.48 ± 0.24	0.60 ± 0.19
AR_0.4_	0.36 ± 0.39	0.36 ± 0.39	0.37 ± 0.38	0.36 ± 0.40	0.35 ± 0.39	0.38 ± 0.42
AR_0.6_	0.39 ± 0.42	0.39 ± 0.42	0.40 ± 0.41	0.39 ± 0.43	0.37 ± 0.42	0.40 ± 0.43
AR_0.8_	0.68 ± 0.35	0.65 ± 0.37	0.66 ± 0.35	0.74 ± 0.32	0.74 ± 0.32	0.81 ± 0.22
κ_0.2_	0.20 ± 0.13	0.23 ± 0.14	0.16 ± 0.09	0.15 ± 0.10	0.22 ± 0.14	0.23 ± 0.10
*σ*_0.2_/*D_p_*	0.23 ± 0.09	0.22 ± 0.09	0.22 ± 0.08	0.21 ± 0.07	0.25 ± 0.10	0.25 ± 0.10

## Data Availability

The MERRA-2 reanalysis product is available from https://disc.gsfc.nasa.gov/datasets/M2TMNXAER_5.12.4/ (accessed on 11 March 2021). The field campaign data are available upon request to the corresponding author (Tiantao Cheng).

## Supplementary Materials

The following supporting information can be downloaded at: https://www.mdpi.com/article/10.3390/ijerph19159608/s1, Figure S1: Spatial distribution pattern of hourly PM_2.5_ and 12-hr average 850 hPa wind filed during 1–2 November, 22–23 November, and 3–4 December; Table S1: Difference and relative change rate of various parameters in LVEs evolution. The abbreviation for fog-haze event is F-H.
