# Supplementary material for: Fog–Haze Transition and Drivers in the Coastal Region of the Yangtze River Delta
Source: Int J Environ Res Public Health. 2022 Aug 4;19(15):9608. doi: 10.3390/ijerph19159608 (PMC9368322; doi:10.3390/ijerph19159608)
Supplement: Supplementary file 1 [file ijerph-19-09608-s001.zip › ijerph-1824435-supplementary.pdf]

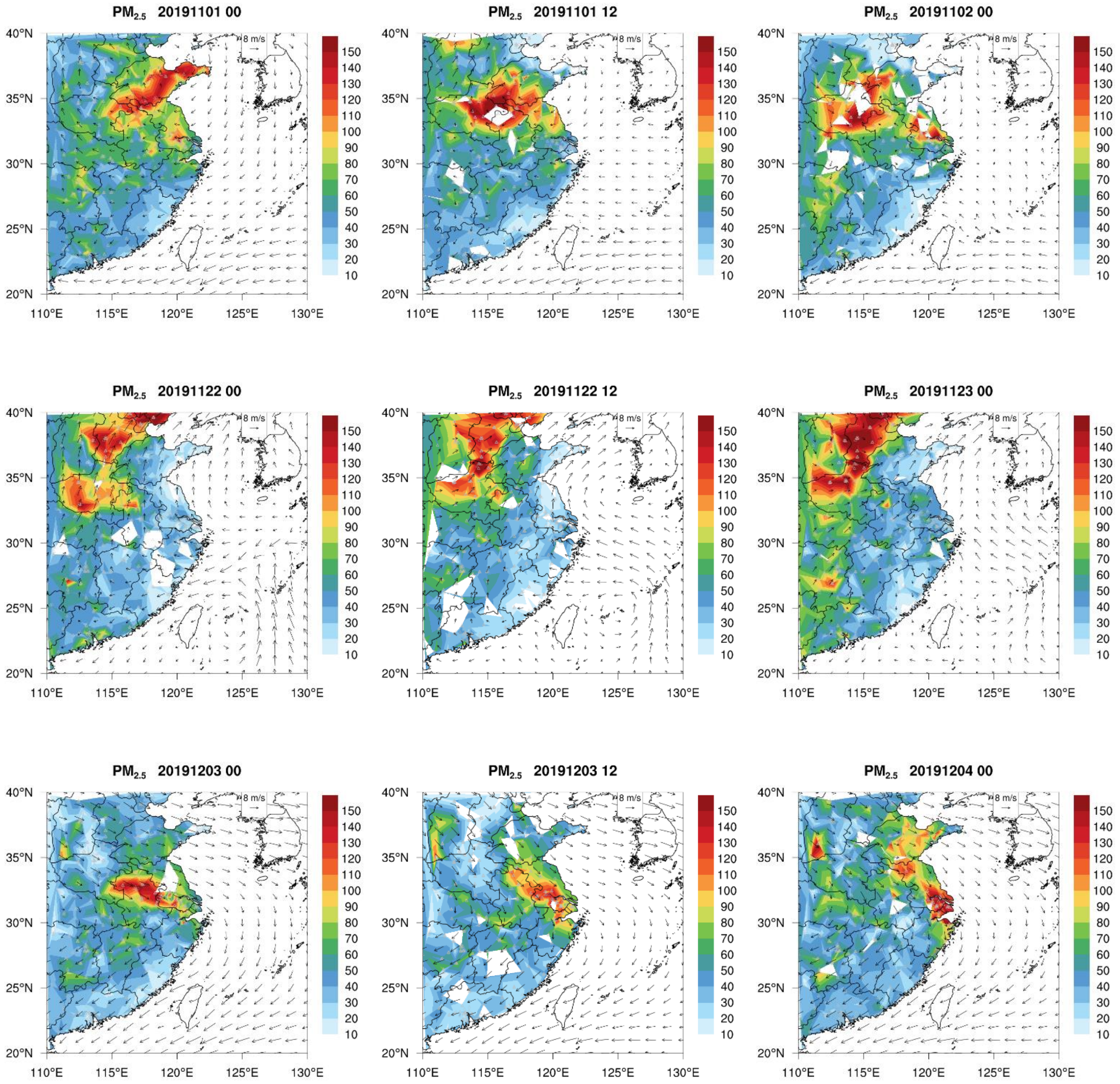

Figure S1. Spatial distribution pattern of hourly $PM_{2.5}$ and 12-hr average 850 hPa wind filed during 1-2 Nov., 22-23 Nov., and 3-4 Dec.

Table S1. Difference and relative change rate of various parameters in LVEs evolution. The abbreviation for fog-haze event is F-H.

	ΔVIS (km)	ΔRH (%)	ΔQ (Rate) (g/kg)	ΔT (Rate) (℃)	ΔLTS (Rate) (℃)	ΔWS （m/s）	ΔN_{CN} (Rate) （cm^{-3}）	$\Delta N_{CCN0.2}$ (Rate) （cm^{-3}）	$\Delta AR_{0.2}$	Δκ	$\Delta\sigma_{0.2}/Dp$
Clean_to_Mist	-5.41	2.00	-0.09 (-1.35%)	-0.50 (-0.18%）	0.07 (0.02%)	0.00	-218.9 (-2.47%)	--	--	--	--
Clean_to_F-H	-4.24	3.00	-0.05 (-0.64%)	-0.80 (-0.27%）	0.32 (0.11%)	-0.20	-140.4 (-1.77%)	808.4 (9.63%)	0.014	0.018	-0.030
Clean_to_Haze	-2.39	8.50	0.28 (7.54%)	-1.20 (-0.43%）	0.40 (0.14%)	-0.55	728.6 (5.96%)	452.9 (5.98%)	0.007	0.007	0.011
Mist_to_Clean	6.89	-7.00	0.22 (3.41%)	1.70 (0.60%）	-0.57 (-0.20%)	0.00	-1459.5 (-29.55%)	-1388.6 (-25.31%)	0.057	0.169	-0.010
Mist_to_Fog	-0.68	0.00	0.00 (0.04%）	-0.00 (-0.00%）	-0.01 (-0.00%)	0.00	-62.5 (-0.44%)	-372.4 (-4.67%)	-0.022	-0.019	-0.008
Mist_to_F-H	0.88	-6.00	0.34 (7.96%)	2.30 (0.80%）	-0.97 (-0.35%)	0.30	-377.3 (-3.55%)	69.0 (3.28%)	-0.014	0.003	0.015
Mist_to_Haze	4.41	-31.00	-0.44 (-5.83%）	4.20 (1.53%）	-1.03 (-0.36%)	1.40	-2479.3 (-28.74%)	-1053.8 (-20.25%)	0.006	0.046	-0.023
Fog_to_Mist	1.10	0.00	0.46 (5.35%)	0.80 (0.28%）	-0.24 (-0.09%)	0.00	-197.5 (-2.29%)	40.3 (0.43%)	-0.003	0.004	-0.019
Fog_to_Haze	4.04	-27.00	-0.32 (0.53%)	4.85 (1.73%）	-0.65 (-0.22%)	1.40	-1271.2 (-11.13%)	-1454.3 (-17.34%)	-0.099	-0.149	0.069
F-H_to_Clean	4.47	-4.5	0.17 (2.52%)	1.40 (0.48%）	0.17 (0.06%)	0.40	-2784.2 (-28.88%)	-301.1 (-25.02%)	0.033	0.025	-0.044
F-H_to_Mist	-0.96	2.00	-0.03 (-0.25%)	-0.45 (-0.16%）	0.10 (0.03%)	-0.15	-317.4 (-1.97%)	-83.4 (-1.12%)	0.000	0.000	-0.008
F-H_to_Fog	-4.99	1.00	-0.91 (-8.12%)	-1.50 (-0.52%）	0.30 (0.11%)	0.30	-394.8 (-3.73%)	--	--	--	--
F-H_to_Haze	0.69	-8.00	-0.08 (-2.08%)	1.20 (0.42%）	-1.04 (-0.37%)	0.60	-1789.6 (-9.04%)	-1017.4 (-8.27%)	0.072	0.016	-0.007
Haze_to_Clean	4.67	-5.00	-0.23 (-2.21%)	1.20 (0.42%）	-0.58 (-0.21%)	0.70	-6.75 (-0.08%)	-77.3 (-2.08%)	0.009	0.016	-0.014
Haze_to_FH	-2.01	10.00	0.08 (2.24%)	-1.40 (-0.51%）	0.23 (0.08%)	-0.20	391.8 (2.93%)	20.1 (0.22%)	0.018	0.023	0.002
